# Multiomics Integration in Skin Diseases with Alterations in Notch Signaling Pathway: PlatOMICs Phase 1 Deployment

**DOI:** 10.3390/ijms22041523

**Published:** 2021-02-03

**Authors:** Lucas André Cavalcanti Brandão, Paola Maura Tricarico, Rossella Gratton, Almerinda Agrelli, Luisa Zupin, Haissam Abou-Saleh, Ronald Moura, Sergio Crovella

**Affiliations:** 1 Department of Advanced Diagnostics, Institute for Maternal and Child Health IRCCS Burlo Garofolo, 34137 Trieste, Italy; lucabrand@gmail.com (L.A.C.B.); rossella.gratton@gmail.com (R.G.); luisa.zupin@burlo.trieste.it (L.Z.); ronaldmoura1989@gmail.com (R.M.); 2 Department of Pathology, Federal University of Pernambuco (UFPE), Recife 1235, Brazil; almerindaapimentel@gmail.com; 3 Department of Biological and Environmental Sciences, College of Arts and Sciences, University of Qatar, Doha 2713, Qatar; hasaleh@qu.edu.qa (H.A.-S.); sgrovella@qu.edu.qa (S.C.)

**Keywords:** omics, genomics, transcriptomics, proteomics, network interaction, skin diseases

## Abstract

The high volume of information produced in the age of omics was and still is an important step to understanding several pathological processes, providing the enlightenment of complex molecular networks and the identification of molecular targets associated with many diseases. Despite these remarkable scientific advances, the majority of the results are disconnected and divergent, making their use limited. Skin diseases with alterations in the Notch signaling pathway were extensively studied during the omics era. In the GWAS Catalog, considering only studies on genomics association (GWAS), several works were deposited, some of which with divergent results. In addition, there are thousands of scientific articles available about these skin diseases. In our study, we focused our attention on skin diseases characterized by the impairment of Notch signaling, this pathway being of pivotal importance in the context of epithelial disorders. We considered the pathologies of five human skin diseases, Hidradenitis Suppurativa, Dowling Degos Disease, Adams–Oliver Syndrome, Psoriasis, and Atopic Dermatitis, in which the molecular alterations in the Notch signaling pathway have been reported. To this end, we started developing a new multiomics platform, PlatOMICs, to integrate and re-analyze omics information, searching for the molecular interactions involved in the pathogenesis of skin diseases with alterations in the Notch signaling pathway.

## 1. Introduction

The skin covers the entire human body’s surface and is considered the largest human organ, with an extension of over two square meters in adult individuals, reaching a weight of 4 kg [1]. The main function of the skin is to act as a physical, immunological, and mechanical barrier that prevents and restricts water (physical) loss and the entry of potential aggressors, such as microorganisms and toxic substances [2]. Therefore, the skin functions as the first immune defense mechanism against infectious agents, a function that is of crucial importance for the maintenance of epithelial microbial diversity since the loss of elements of the microbiota can favor the growth of pathogens and promote epithelial diseases during or after skin infection [3,4].

Moreover, the skin is among the largest sensory organs involved in the transduction of multiple environmental stimuli, such as pressure and temperature, to the central nervous system, thus evoking appropriate physiological responses. In addition, the epithelial tissue is responsible for the production of several inflammatory cytokines that allow the regulation of many homeostatic functions [1,5]. The breakdown of skin homeostasis leads to numerous skin disorders affecting the quality of life and occasionally leading to death. Homeostatic mismatch may be caused by exogenous agents’ actions (environmental factors and infections) or by endogenous events (genetic or hormonal changes). However, most studies on skin pathogenesis are carried out focusing on a specific cell or molecule. Such studies bring the expectation of understanding how a cell or a molecule deregulates tissue homeostasis, although the understanding of the tissue microenvironment as a whole is essential to fully comprehend pathological processes.

Interactions between the molecules of the epithelial tissue microenvironment produced by the immune regulatory cells (cells of the epithelium and stroma) are crucial for maintaining the balance between the tissue, the microbiota, and the environment [3,6,7]. Despite this aspect, many skin diseases remain without defined pathogenesis and, consequently, without an adequate diagnosis and treatment.

Within the latter category, we can include skin diseases presenting alterations in Notch signaling. For these disorders, in spite of knowing the pathway primarily involved (Notch signaling), the etiopathogenesis is far from being completely understood. This lack of knowledge leads to a direct repercussion in selecting appropriate therapeutic strategies, which are still only supportive and not yet tailored to the disease and patients’ needs.

Thus, our goal is to refine the data available in the scientific literature and omics repositories on those skin diseases characterized by defects in Notch signaling, seeking to describe networks of molecular interactions in the epithelial tissue potentially involved in the loss of homeostasis in this district, an event that may lead to the onset of different skin pathologies.

In this context, we developed an omics platform (PlatOMICs) for skin studies that compiles a set of tools and bioinformatics applications that can allow the retrieval of scientific literature data (genomics, epigenomics, transcriptomics, proteomics, and microbiomics) together with the analysis, deciphering, interpretation, and integration of all these sets of information automatically, therefore building networks of molecular interactions and omics meta-analysis.

Intending to search for similarities between different skin disorders and their pathogenic mechanisms, we traced integrated molecular models that consider different etiological agents, both endogenous and exogenous, that are known to deregulate the homeostasis of epithelial tissues. Specifically, we initially assumed that genetic variants might alter the functioning of metabolic pathways, an action that can range from the production of mRNA and miRNA to the interactions between translated proteins. We also considered the pivotal role of environmental interactions in the skin, mainly driven by microbiota, as another critical aspect to be examined while dealing with skin diseases. The usage of this approach allows a broad characterization of the molecular mechanisms and a deep identification of the role exerted by the molecular pathway as a whole in the context of skin pathologies. This integrative outlook may contribute to the recognition of new diagnostic targets, to the enhancement of the prognosis, and to the development of novel and appropriate therapeutic options.

## 2. Multiomics Integration Applied to Skin Diseases with Notch Signaling Alterations

Notch signaling plays a key role in maintaining the homeostasis of the skin by balancing and regulating keratinocyte proliferation, progressive differentiation by finely modulating the advancement of keratinocytes through the various layers of the skin, migration, and apoptosis, both during normal development and pathological conditions [8]. An aberrant progression of Notch signaling, either due to anomalous regulation or direct mutations, can induce skin diseases [9]. To date, molecular alterations in Notch signaling pathway have been reported for five human skin diseases, Hidradenitis Suppurativa (HS), Dowling Degos Disease (DDD), Adams–Oliver Syndrome (AOS), Psoriasis (PS), and Atopic Dermatitis (AD) [10]. Therefore, a thorough characterization of this signaling route seems to be promising in unraveling potential novel pathogenic scenarios involved in these skin disorders. Considering this crucial aspect, in order to further restrict the search, we decided to consider in this study only the skin diseases possessing alterations in the Notch pathway, excluding malignancies.

In the last five years, there have been 1555 articles regarding these five diseases and omics (genomics, transcriptomics, proteomics, and microbiomics) studies available in PubMed, last accessed on 18 November 2020 [11].

Specifically, considering these five skin disorders known to possess alterations in Notch signaling, 821 articles about the genome, 225 about the transcriptome, 143 about the proteome, and 602 about the microbiome were published (Table 1).

An important aspect that needs to be considered is that all of these five diseases are multifactorial disorders, and for this reason, an integrated approach based on multiomics integration is strongly required.

If we search for studies performed in these five diseases employing a multiomics approach, we retrieve only 11 articles in PubMed published in the last five years [12].

It is interesting to note that these 11 articles examine only two of the five researched diseases: AD and PS (Table 1). Amongst the 11 retrieved articles, four are reviews [8,13,14,15], one is a protocol for a case-control study [16], and one is not a multiomics study [17]. These papers were not taken into account for further processing. Therefore, only 5 papers were further considered [18,19,20,21,22]. None of these articles includes DDD or AOS; thus, nowadays, the multiomics perspective has not been used for both diseases.

AD is defined as one of the most frequent pruritic, chronic, and inflammatory disorders affecting approximately 20% of adolescents and children worldwide, yet possessing a lower incidence in the adult population, ranging between 2–17% [23]. The pathogenesis of AD is complex and multifactorial; indeed, a combination of genetic predisposition, skin barrier abnormalities, and immune dysregulation, the latter principally given by an excessively enhanced T helper-2 activity, seems to have a strong impact on the onset, progression, and severity of the disease [24,25].

Soren Mucha and collaborators observed a major role of rare coding variants in AD acting independently of common variants. They used a multiomics approach analyzing low-frequency and rare protein-coding variants using an exome chip and replication genotype data of 15,574 AD with respect to 377,839 control subjects, combined with the whole-transcriptome of lesional, nonlesional, and healthy skin samples of 27 AD with respect to 38 control subjects. They observed that the association signals of low-frequency and rare coding variants are enriched in 5 genes of the IL13 pathway and also that a pivotal role in the AD regulatory signaling pathway is played by CD200/CD2OOR1/DOK2 signaling [22].

However, no extensive proteomic analysis has been performed allowing the integration of the findings obtained, thus limiting the possibility of comparison with other multiomics studies.

Donald Y. M. Leung and collaborators observed that the nonlesional skin surface in patients with AD and food allergy has unique properties, a unique composition of skin proteins, lipids, and RNA transcripts, caused by an immature skin barrier and type 2 immune activation. These observations are based on multiomics approaches analyzing transepidermal water loss, stratum corneum composition, the skin transcriptome, the skin proteome, and the skin microbiome of 62 subjects, 21 patients with AD and food allergy, 19 patients with AD and without food allergy, and 22 healthy subjects [18]. Nevertheless, in this study, the authors did not cover omics entirely, since genomic profiling of all patients was lacking, rendering the retrieved results focused on just a particular aspect of the disease.

Le Duc Huy Ta and collaborators described an aberrant developmental trajectory of the gut microbiome and stool metabolome in AD allergen-sensitized infants. They performed multiomics studies (meta-genomics, meta-transcriptomics, and meta-metabolomics) in 63 infants between 3 weeks and 12 months of age and compared them with AD and controls. This multiomics approach aims to better characterize and identify potential microbial pathways involved in the early onset of AD in infants. Their results suggest that in infants affected by AD and allergen sensitization, the metabolome and gut microbiome are abnormal within the first three months of age. These alterations have been hypothesized to entail a decrement in propionate and butyrate-producing microorganisms [21]. However, the impact of the genetic background of the enrolled subjects has not been evaluated, with no consequent possibility of integrating the findings with the host genetic profile.

PS is a chronic, immune-mediated inflammatory disorder characterized by primary cutaneous manifestations and possesses an estimated incidence of 14 in 10,000 persons per year [26]. Additionally, PS is known to have multifactorial pathogenesis depending on the combination of complex cross-talks between the genetic background, changes in the skin’s microbiome, and disturbances in the adaptive and innate immune responses related primarily to the epidermal district [27]. The principal events observed in PS cases comprise an anomalous differentiation and proliferation of keratinocytes that ultimately lead to dermal invasion by immune cells, hyperplasia of the epidermis, and increased permeability of large-caliber vessels [28].

Junqin Li and collaborators confirm the contribution of the de novo mutations in PS. In particular, they performed a multiomics study in 8 monozygotic twin pairs discordant for PS analyzing whole-genome sequencing, RNA-sequencing for CD4^+^, CD8^+^ T cells, and for lesion and nonlesion skin. They reported a de novo loss of function mutation in the *C3* gene [20]. The findings are of great interest, but it lacked a comparison with other ethnic groups and a proteomic validation of the results to allow a comparison with other multiomics studies.

PS presents a complex etiopathogenesis in which the interplay between genetic background and environmental factors seems crucial; nevertheless, the exact molecular actors and mechanisms underlying these events still need to be fully characterized. In order to obtain a general and more complete understanding of the major cellular processes, networks, and genes principally implied in the onset, progression, and risk of PS, Zhao Yuqi and collaborators performed a study that provides the integration of multiple multiomics datasets, such as epigenome-wide association studies (EWAS), genome-wide association studies (GWAS), expression quantitative trait loci, tissue-specific transcriptomics, gene networks, and biological pathway analysis to determine potential targets that are epigenetically and genetically associated with PS risk. The output of these results provided a reconfirmation of some of the major pathways already known to correlate with the PS phenotype, including the IL-17 pathway, natural killer T cell function, NO2-dependent, IL-12 pathway, Th1/Th2 axis, JAK/STAT signaling, and cytokine and chemokine signaling, all of which were also confirmed in this work to have high enrichment values in terms of associated epigenetic and genetic variants. Additionally, other new processes for PS were retrieved and include proteasome pathways, lipid metabolism, insulin signaling, collagen synthesis, adipokine signaling, cell-to-cell communication routes, and endoplasmic reticulum phagosome formation [19]. The integrated computational approach used was very effective; however, it did not consider all omics possibly involved in the disease, such as the microbiome or proteome, thus resulting in a partial story of PS etiopathogenesis.

The five abovementioned multiomics studies, despite having provided novel valuable insights into the etiopathogenesis of AD and PS, are suffering from some intrinsic limitations related to data integration within the studies due to the different approaches used to obtain the results. These limitations are not dealing with the scientific value of the studies, which are relevant but are related to the different methodological strategies employed, so the need for omics data uniformity and integration is an important issue to consider.

## 3. Perspectives on Multiomics Integration for Skin Diseases with Alterations in the Notch Signaling Pathway

The use of multiomics approaches to reveal mechanisms involved in various disorders, new biomarkers, or other features (i.e., drug design) has been rapidly growing in the last few years. Around 3500 articles have been produced since 2005 (multiomics OR multiomics), 1400 articles being tagged as human studies.

Regarding the skin, 54 studies (10 reviews) on human skin multiomics are found on PubMed ((multiomics OR multiomics) AND skin), the microbiome, genome, methylome, and transcriptome being the most common ones.

Multiomics integration has been proposed as a combination of computational methodologies able to connect, for instance, the genome and transcriptome findings in complex multiomics network results. From our perspective, the current approach needs to interrogate and iterate the literature and the databases of previous molecular and omics studies, with the further aim of using this information to perform a new multiomics study integration.

Based on these necessities, we started the development of a new platform called PlatOMICs, to be employed for the Special Issue “OMICs, Data Integration, and Applications in Personalized Medicine” in the *International Journal of Molecular Science*, considering skin diseases with alterations in the Notch signaling pathway as an initial model [29].

### 3.1. New Multiomics Platform (PlatOMICs)

Currently, the omics platform (PlatOMICs) is under deployment in international cooperation, including in Brazil, Qatar, and Italy. PlatOMICs will be an online platform offering services to access and analyze the scientific literature and omics data automatically with high accuracy. The deployment was divided into three phases, and in the present work, we report phase 1.

**Figure 1:**
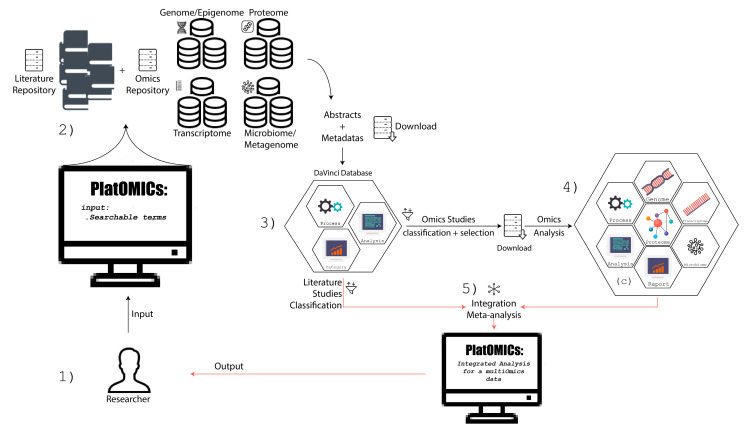
Workflow of omics Platform (PlatOMICs) for omics integration. (**1**). The user informs the descriptors, categorical terms, and keywords in PlatOMICs. (**2**,**3**) Through DaVinci tool, the literature and the omics databases will be evaluated. (**4**) Selected omics studies and new ones are (re)analyzed and integrated by standard pipelines. (**5**) PlatOMICs produces final meta-analysis–multiomics integration results in a friendly interface.

The overall PlatOMICs workflow is provided in Figure 1.

Briefly, PlatOMICs multiomics analysis is divided into three phases: first, the whole available literature and omics databases will be interrogated and analyzed; second, previous and new omics (or multiomics) studies will be analyzed and integrated; third, both findings will finally compose the ultimate multiomics integration in a meta-multiomics analysis.

The first analysis (Phase I) outputted by PlatOMICs is performed by the new tool called DaVinci Literature and Database Analysis (under submission and not publicly available). In brief, DaVinci is able to scan several databases, such as PubMed, SRA, GEODatabase, and GWAS Catalog, extracting multiple information from the summary, abstracts, and other meta-data information to report a syntax analysis and molecular panels (genes, variants, tissues, cells, and drugs). Next, following the study and sample selection from previous research, raw data could be downloaded. The analysis, including the new omics (or multiomics) when performed, will be carried out by the same standard pipeline, thus ensuring a more reliable and homogenous analysis. Therefore, PlatOMICs will contain the results obtained and integrated from the literature, databases, and new omics studies.

### 3.2. Phase 1 of PlatOMICs for Skin Diseases with Alterations in the Notch Signaling Pathway

#### 3.2.1. Analysis from the Literature: Molecular Insights to Multiomics Integration

The literature scan was accomplished by assessing the following term “(Hidradenitis Suppurativa OR Dowling Degos Disease OR Adams Oliver Syndrome OR Psoriasis OR Atopic Dermatitis) AND (Genome OR transcriptome OR proteome OR epigenome OR microbiome OR metagenome OR metabolome OR omic OR multi-omic) AND ‘Homo sapiens’ (orgn:__txid9606)” using the DaVinci tool.

A DaVinci literature database (DaVinci Lit) was created with 1252 articles retrieved from PubMed; 82 were excluded due to the absence of an abstract/summary. The 1170 remaining articles were analyzed, classified, and categorized.

Table 2 shows the word atomization results from the abstracts of DaVinci Lit. As expected, the most cited words were “skin” and “patient”. The words immune, inflammatory, and inflammation were common. In the 742 (63.4%) articles, at least once, one of these words was cited. We sought the context of each of these words, revealing they were mainly used to explain the immune and inflammatory conditions of the disorders. “Expression” was cited along 333 (28.4%) articles to demonstrate molecular expression on experimental works of the transcriptome (48), epigenome or methylome (36), and proteome (12). The last word worth commenting on is “gut”. Gut was present in 158 articles and refers to the existing relationship between gut dysbiosis and allergic onset (also included in the top-cited words) disbalance.

The overview of Table 2 enabled us to understand the main focus of the omics literature on skin diseases with alterations in the Notch signaling pathway. Then, we categorized the whole DaVinci Lit into five classes of omics (Table 3). Most of the articles were included as a genome or microbiome (metagenome) study, followed by a transcriptome and multiomics approach. In the multiomics category, the most used omics approaches included genome plus microbiome and genome plus transcriptome.

The next step in PlatOMICs is to extract the genes and variants from the literature. The inception is to decrypt the genes/variant previously known to be involved with skin diseases with alterations in the Notch signaling pathway. The gene atomization process retrieves 546 genes. From these genes, each time, we extracted the context in which the gene was cited. In total, 465 articles and 1308 gene contexts were analyzed. Subsequently, four researchers classified, independently, the gene relations as associated or not with the disease. Of these, 80 genes were excluded, and 426 genes were associated (Supplementary Materials Table S1).

Next, PlatOMICs outputted the top 10 pathways and gene ontology (GO) predicted by these genes (Table 4).

Enrichment pathway and GO analysis were conducted by the reactomePA, limma, and topGO Bioconductor package. The pathway reveals the role of interleukin (IL) signaling, mainly driven by IL-4, IL-13, and IL-10. GO adds the defense response and interspecies interactions between organisms. Both descriptions point out inflammation and skin microbial host defense as key outcomes from the global literature findings, suggesting that these pathways and GO should be included in future omics studies.

Drug analysis described the top drugs cited in omics articles related to skin diseases with alterations in the Notch signaling pathway: imiquimod, dupilumab, adalimumab, ustekinumab, methotrexate, secukinumab, etanercept, infliximab, and cyclosporine. In general, these drugs were used to induce skin lesions in animal models, for in vitro experimental analysis or administered to patients.

#### 3.2.2. Analysis from Databases: Omics and Multiomics Discovery

The Omics database scan was performed using the following term “(Hidradenitis Suppurativa OR Dowling Degos Disease OR Adams Oliver Syndrome OR Psoriasis OR Atopic Dermatitis) AND ‘Homo sapiens’ (orgn:__txid9606)” using the DaVinci tool. We included the GEO, SRA, Bioproject, Biosamples, and GWAS Catalog databases.

PlatOMICs creates a DaVinci Omics database (DaVinci Omics), including 369 omics studies. After a human curation, 25 studies were excluded, to not be related to one of the five diseases. Therefore, 344 omics studies remained (Table 5 and Supplementary Materials Table S2); 319 were sourced from Bioproject or GEO, and 45 from the GWAS catalog. The majority of the studies were related to transcriptome and genome analysis.

PlatOMICs also performed gene atomization on DaVinci Omics. This analysis accrued 158 genes, most of which were found to be similar to the DaVinci Lit output. Equals enriched pathways and GO from Table 4 were found, thereby ratifying the importance of these pathways and GO in multiomics integration. The most cited drugs in omics studies were etanercept, ustekinumab, tofacitinib, secukinumab, dupilumab, adalimumab, and infliximab.

In total, 2742 public BioSamples were found from 75 BioProjects, and 32,003 samples were retrieved in 184 GEO series, totaling 34,745 samples.

### 3.3. Phase 2 and 3 of PlatOMICs for Skin Diseases with Alterations in the Notch Signaling Pathway

Phase 2 of PlatOMICs development is directed to re-analyze 34,745 samples from the DaVinci Omics database. Our team is implementing an algorithm in order to curate, classify, and download selected samples. The approach will allow us to re-run the raw sequence or chip data from scratch, avoiding normalization differences and diminishing heterogeneity, and promoting the preparation for the third PlatOMICs phase, namely, the meta-analysis–omics integration. All omics analyses will be realized by a standard multiomics pipeline currently in progress in our laboratory. All of these studies will be analyzed, according to the previous classification in their omics group, by a meta-analysis by models of random effects and classified according to their characteristics [30].

During phase 3, the multiomics integration will be performed. In this phase, literature information and previous omics studies will be investigated, connected, and integrated. Multiple layers of omics integrations are being developed by our team.

After all phases are complete, the PlatOMICs will be able to connect all the obtained information by expanding or directing the perspectives of the gained results. As an example of this operation, we can describe that after DaVinci analysis, an important pathway or molecule related to the development of a pathological event was pointed out.

## 4. Conclusions

The scientific goal of PlatOMICs is to help understand the complex molecular networks using existing data. The use of this cost-efficient and time-saving methodology can help enlighten the most diverse biological mechanisms and guide researchers to elaborate more straightened hypotheses.

It is expected that the software assists the scientific community in omics analysis and meta-analyses from previously published data.

Due to the advances in molecular science techniques, it is possible to study the entire set of molecules present in a cell or tissue. This growing field, commonly called omics, refers to studies of the entire genome, transcriptome, or proteome, etc. On this basis, the amount of information generated by each experiment is remarkable. For example, there are about 20,000 genes and millions of variants of these genes in humans. In addition, each cell type has a specific pattern of expression of proteins, depending on the microenvironment conditions at a given time.

Consequently, the great access to molecular techniques was not accompanied by data processing, analysis, or integration tools. A few bioinformatics tools for genomic, transcriptomic, proteomic, epigenomic, and microbiome analyses have an intuitive interface and usually require advanced knowledge in programming languages. To date, there is no single open-source program to perform end-to-end analysis, requiring several applications to perform a reliable analysis. In addition, the number of published scientific articles is growing exponentially, making it impossible to process this amount of information without computer assistance.

The accumulation of scientific texts and omics data deposited in the databases may never have been correlated and analyzed in conjunction. In this perspective, significant scientific responses may have been produced but are still uncovered. Thus, PlatOMICs was developed to help analyze these available omics data. In the presented work, we applied PlatOMICs in the study of skin diseases as a validation model.

## Figures and Tables

**Table 1:** Scientific publications from 2016 to 2020 on Notch dermatological diseases, excluding bibliographic reviews and meta-analyses.

Omics	Total	HS	DDD	AOS	PS	AD
Genome	821	37	5	9	519	290
Transcriptome	225	14	0	0	150	89
Proteome	143	8	0	0	84	61
Microbiome	602	39	0	0	180	433
Multiomics	11	0	0	0	4	7

**Table 2:** The top 30 cited atomization results from the omics literature concerning skin diseases with alterations in the Notch signaling pathway.

N	Word	N	Word	N	Word
1	skin	11	microbiota	21	response
2	patients	12	clinical	22	mice
3	expression	13	aureus	23	controls
4	associated	14	development	24	potential
5	cell/cells	15	healthy	25	levels
6	studies	16	allergic	26	variants
7	diseases	17	pathogenesis	27	microbial
8	immune	18	gut	28	group
9	inflammatory/inflammation	19	barrier	29	protein
10	treatment	20	chronic	30	children

**Table 3:** Omics categorization of the literature from omics studies concerning skin diseases with alterations in the Notch signaling pathway.

Category	Number of Articles
genome	245
microbiome	241
transcriptome	97
proteome	32
metabolome	2
Multiomics	95

**Table 4:** Top 10 enriched pathways and a gene ontology of 426 genes associated with skin diseases with alterations in the Notch signaling pathway.

Reactome ID	Pathway Description	GO ID	GO Description
R-HSA-449147	Signaling by Interleukins	GO:0034097	Response to cytokine
R-HSA-6785807	Interleukin-4 and Interleukin-13 signaling	GO:0019221	Cytokine-mediated signaling pathway
R-HSA-6783783	Interleukin-10 signaling	GO:0071345	Cellular response to cytokine stimulus
R-HSA-877300	Interferon gamma signaling	GO:0002376	Immune system process
R-HSA-447115	Interleukin-12 family signaling	GO:0006952	Defense response
R-HSA-8854691	Interleukin-20 family signaling	GO:0009605	Response to external stimulus
R-HSA-913531	Interferon Signaling	GO:0044419	Interspecies interaction between organisms
R-HSA-380108	Chemokine receptors bind chemokines	GO:0070887	Cellular response to chemical stimulus
R-HSA-451927	Interleukin-2 family signaling	GO:0010033	Response to organic substance
R-HSA-1059683	Interleukin-6 signaling	GO:0051707	Response to other organisms

**Table 5:** Numbers of omics studies according to omics type and disorder with alterations in the Notch signaling pathway.

Omics Type	Number of Omics Studies				
	Global	Atopic Dermatitis	Hidradenitis Suppurativa	Psoriasis	Atopic Dermatitis and Psoriasis
Transcriptome	212	45	12	141	14
Genome	71	14	2	51	4
Microbiome	8	2	2	3	1
Proteome	2	0	0	2	0
Genome and transcriptome	44	12	0	26	6
Transcriptome and proteome	3	1	0	2	0
Transcriptome and microbiome	2	1	0	1	0
Genome and microbiome	1	1	0	0	0
Proteome and microbiome	1	0	0	1	0
Total	344	76	16	227	25

## Supplementary Materials

The following are available online at https://www.mdpi.com/1422-0067/22/4/1523/s1, Table S1: List of genes associated in skin diseases with alterations in the Notch signaling pathway; Table S2: List of studies and project retrieved from omics databases involved in skin diseases with alterations in the Notch signaling pathway.
